# Supplementary material for: Identification of photoperiod-induced specific miRNAs in the adrenal glands of Sunite sheep (Ovis aries)
Source: Front Vet Sci. 2022 Jul 22;9:888207. doi: 10.3389/fvets.2022.888207 (PMC9354845; doi:10.3389/fvets.2022.888207)
Supplement: Supplementary file 1 [file Data_Sheet_1.ZIP › Supplementary file/miRDeep2 file.zip!/miRDeep2 file/Novel_Structure/SPLP42A1/Novel_69.pdf]

Provisional ID	: NC_019476.2_25470
Score total	: 2.4
Score for star read(s)	: -1.3
Score for read counts	: 0
Score for mfe	: 2.1
Score for randfold	: 1.6
Score for cons. seed	:
Total read count	: 142
Mature read count	: 132
Loop read count	: 0
Star read count	: 10

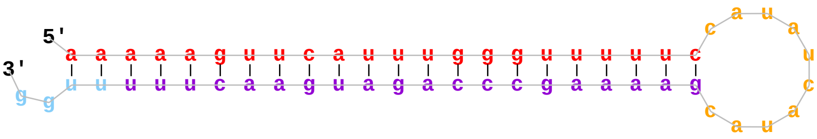

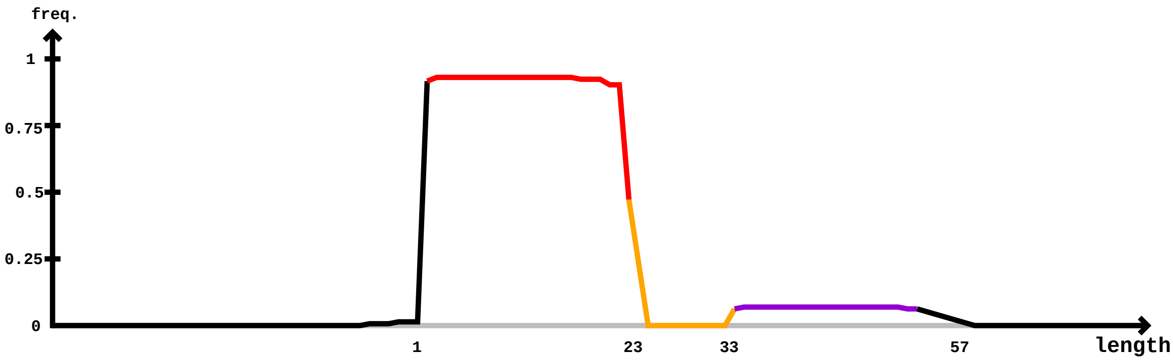

Mature Star

```
5'- gagcaggagaagcauacaaauagguauuggguuggucaaaaaguucauuuggguuuuuccauaucauacgaaaagcccagaugaacuuuuuggucaauccaguauuaaaacc  -3'  obs
    gagcaggagaagcauacaaauagguauuggguuggucaaaaaguucauuuggguuuuuccauaucauacgaaaagcccagaugaacuuuuuggucaauccaguauuaaaacc       exp
    ..((.......)).........(((((((((((((((((((((((((((((((((((((..........)))))))))))))))))))))))))))))))))))))......  reads  mm  sample
    ...............................uuggGcaaaaaguucauuuggg...........................................................  1  1  seq
    ..................................gucaaaaaAuucauuuggguuu........................................................  1  1  seq
    .....................................aaaaaguucauuuggguuu........................................................  1  0  seq
    .....................................aaaaaguucGuuuggguuuuu......................................................  62  1  seq
    .....................................aaaaaguucGuuuggguuuuuc.....................................................  66  1  seq
    .....................................aaaaaguucauuuggguuuuucU....................................................  1  1  seq
    ......................................Uaaaguucauuuggguuu........................................................  1  1  seq
    ......................................aaaaguucauuuggguuuuucU....................................................  1  1  seq
    .....................................................................gaaaaAcccagaugaacu.........................  1  1  seq
    .....................................................................gaaaaAcccagaugaacuuu.......................  8  1  seq
    ......................................................................aaaagcccagaugaacuuuuug....................  1  0  seq
```
